# Retraction: Efficacy and safety of tripterygium glycosides for active moderate to severe Graves’ ophthalmopathy: a randomised, observer-masked, single-centre trial

**DOI:** 10.1530/EJE-20-0857z

**Published:** 2021-04-01

**Authors:** Xiaozhen Ye, Heng Zhao, Jun Liu, Bin Lu, Jiaqing Shao, Jian Wang

**Affiliations:** 1 Department of Endocrinology, Jinling Hospital, Affiliated Hospital of Medical College of Nanjing University; 2 Department of Endocrinology, Taikang Xianlin Drum Tower Hospital, Nanjing, Jiangsu Province, China

This article has been retracted at the request of the authors:

Ye X, Zhao H, Liu J, Lu B, Shao J & Wang J. Efficacy and safety of tripterygium glycosides for active moderate to severe Graves’ ophthalmopathy: a randomised, observer-masked, single-centre trial. *European Journal of Endocrinology* **184** 277–287. (https://doi.org/10.1530/EJE-20-0857)

The article has been retracted after the discovery of a serious error with the data used in the analysis which rendered the results and conclusions of this article invalid. As such, and with agreement from all authors, the article has now been retracted.

This request to retract the article was received on 9 March 2021 and the retraction notice was published on 12 March 2021.

